# Identification of novel pathogenic *ABCA4* variants in a Han Chinese family with Stargardt disease

**DOI:** 10.1042/BSR20180872

**Published:** 2019-01-15

**Authors:** Qin Xiang, Yanna Cao, Hongbo Xu, Yi Guo, Zhijian Yang, Lu Xu, Lamei Yuan, Hao Deng

**Affiliations:** 1 Postdoctoral Research Station of Basic Medicine, The Third Xiangya Hospital, Central South University, Changsha, China; 2 Center for Experimental Medicine, The Third Xiangya Hospital, Central South University, Changsha, China; 3 Department of Ophthalmology, The Third Xiangya Hospital, Central South University, Changsha, China; 4 Department of Medical Information, Information Security and Big Data Research Institute, Central South University, Changsha, China

**Keywords:** ABCA4, missense variant, Stargardt disease, whole exome sequencing

## Abstract

Stargardt disease (STGD1, OMIM 248200) is a common hereditary juvenile or early adult onset macular degeneration. It ultimately leads to progressive central vision loss. Here, we sought to identify gene mutations associated with STGD1 in a three-generation Han Chinese pedigree by whole exome sequencing and Sanger sequencing. Two novel potentially pathogenic variants in a compound heterozygous state, c.3607G>T (p.(Gly1203Trp)) and c.6722T>C (p.(Leu2241Pro)), in the ATP binding cassette subfamily A member 4 gene (*ABCA4*) were identified as contributing to the family’s STGD1 phenotype. These variants may impact the ABCA4 protein structure and reduce the retinal-activated ATPase activity, leading to abnormal *all-trans* retinal accumulation in photoreceptor outer segments and in retinal pigment epithelium cells. The present study broadens the mutational spectrum of the *ABCA4* responsible for STGD1. A combination of whole exome sequencing and Sanger sequencing is likely to be a time-saving and cost-efficient approach to screen pathogenic variants in genetic disorders caused by sizable genes, as well as avoiding misdiagnosis. These results perhaps refine genetic counseling and *ABCA4*-targetted treatments for families affected by STGD1.

## Introduction

Stargardt disease (STGD1, OMIM 248200), also known as Stargardt’s macular dystrophy or juvenile macular degeneration, was initially described by Karl Stargardt in 1909 [1,2]. It accounts for approximately 7% of all congenital retinal disorders [3]. The peak onset age for STGD1 is childhood or early adulthood with a prevalence of 0.1–0.125‰ worldwide [3,4]. STGD1 patients primarily present with progressive central vision loss, retinal pigment epithelium (RPE) atrophy, a beaten-bronze display, and yellowish flecks, associated with lipofuscin accumulation in maculae and/or central and peripheral areas of the retina [4–6]. Histologically, it is primarily related to a pervasive lipofuscin deposition in RPE, toxic metabolite accumulation, and excessive photoreceptor cell death [7]. STGD1 is always inherited as an autosomal recessive (AR) form caused by the ATP binding cassette subfamily A member 4 gene (*ABCA4*) mutations, although autosomal dominant STGD1-like phenotypes have been reported to be associated with mutations in the ELOVL fatty acid elongase 4 gene (*ELOVL4*), the prominin 1 gene (*PROM1*), the peripherin 2 gene (*PRPH2*), the bestrophin 1 gene (*BEST1*), and the crumbs 1 cell polarity complex component gene (*CRB1*) [8–12].

The photoreceptor cell-specific ABCA4 protein encoded by the *ABCA4* is an important membrane protein composed of two exocytoplasmic domains, two transmembrane domains, and two cytoplasmic domains, which transport *all-trans* retinal, a by-product of the retinoid cycle, from intra-disc space to photoreceptor cytoplasm to avoid abnormal accumulation of *all-trans* retinal in photoreceptors outer segments and in RPE cells [13,14]. As an N-retinylidene-phosphatidylethanolamine (NRPE) transporter, the protein is mainly involved in ATP-dependent vitamin A metabolite translocation from a photoreceptor outer segment disc lumen to cytoplasm [3,14]. *ABCA4* mutations primarily generate dysfunctional ABCA4 proteins (Rim proteins) resulting in photoreceptor disruption and degeneration [15]. Currently, over 800 mutations in *ABCA4*, including missense, nonsense, small insertion and deletion, frameshift, and splicing, have been reported as causing STGD1 [16]. Genetic analysis has revealed that compound heterozygous or homozygous mutations in *ABCA4* are implicated in STGD1 [17–19].

Next-generation sequencing technology has become a low cost and shorter time tool nowadays for screening pathogenic mutations of hereditary diseases [20]. Using whole exome sequencing combined with Sanger sequencing, the present study identified two novel potentially pathogenic variants in a compound heterozygous state, c.3607G>T (p.(Gly1203Trp)) and c.6722T>C (p.(Leu2241Pro)), in *ABCA4* of a Han Chinese family affected by STGD1, and also described associated clinical phenotypes.

## Materials and methods

### Subjects and clinical assessment

A three-generation, seven-member Han Chinese pedigree with STGD1 and 100 ethnically matched independent unaffected individuals (50 males and 50 females, age: 35.7 ± 2.8 years) were recruited for the present study. Ophthalmologic examinations were performed on all available individuals by two experienced ophthalmologists. The examinations included best corrected visual acuity (BCVA) assessed by the Snellen visual chart, slit-lamp biomicroscopy, microperimetry, fundus examination, fundus fluorescein angiography (FFA), and optical coherence tomography (OCT). All clinical diagnoses for STGD1 phenotype were based upon a family history of recessive inheritance, first or second decade onset, progressive bilateral central vision loss, macular atrophy or dystrophy with a beaten-bronze appearance, retinal yellowish flecks, and a lack of pigmented bone spicules [19,21]. This research was permitted by the Ethical Committee of the Third Xiangya Hospital of Central South University, Changsha, Hunan, P.R. China, and adhered to the Declaration of Helsinki principles. Written informed consent was obtained from each subject or their legal guardians. Peripheral blood was drawn from six family members. Genomic DNA (gDNA) was extracted from blood using a phenol-chloroform extraction strategy as previously described [22,23].

### Whole exome sequencing

Whole exome sequencing was performed on the proband from the pedigree with STGD1. The gDNA of the proband was assessed for integrity using agarose gel electrophoresis and quantitated using NanoDrop 2000 (Thermo Fisher Scientific, Waltham, U.S.A.). Approximately 3.0 µg of gDNA was sheared into fragments. The 3′ end of DNA fragments were then ligated with an ‘A’ to repair. Illumina adapters were added to the DNA fragments and final 350–450 base pairs including adapter products were applied to PCR amplification. The ultimate products were confirmed using an Agilent 2100 Bioanalyzer system (Agilent Technologies Inc., Santa Clara, U.S.A.). Protein coding regions in human gDNA were captured by an Agilent SureSelect Human All Exon V6 Kit (Agilent Technologies Inc., Santa Clara, U.S.A.) following the manufacturer’s instructions. Exome libraries were sequenced on a HiSeq 2000 platform (Illumina Inc., San Diego, U.S.A.) by a commercial service from Novogene Bioinformatics Institute (Beijing, China) in accordance with the manufacturer’s protocols.

### Variants calling and validation

After base calling, low-quality and adapter reads were removed from raw reads using Solexa QA package and Cutadapt program, respectively. Effective reads were mapped on to the human reference genome UCSC (GRCh37/hg19) with a Burrows–Wheeler Aligner (BWA) (http://bio-bwa.sourceforge.net/) [24]. SAMtools (http://samtools.sourceforge.net/) and Picard tools (http://sourceforge.net/projects/picard/) were utilized to rank sequence alignment results and remove duplicate reads, respectively. Genome Analysis Toolkit program (http://software.broadinstitute.org/gatk/) was used to realign and detect single nucleotide polymorphism (SNP) and insertion/deletion (Indel) variations. SNPs and Indels were identified with SAMtools and annotated with ANNOVAR (ANNOtate VARiation) software (http://annovar.openbioinformatics.org/). The variants identified above were filtered against the following four databases: 1000 Genomes Project (1000G_all, http://www.internationalgenome.org/), Single Nucleotide Polymorphism Database 147 (dbSNP147, http://www.ncbi.nlm.nih.gov/projects/SNP/), Exome Aggregation Consortium (ExAC_EAS, http://exac.broadinstitute.org), and National Heart, Lung and Blood Institute Exome Sequencing Project (ESP6500_all, http://evs.gs.washington.edu/EVS/). Remaining variants with a minor allele frequency <0.001 were further processed according to the 700 Han Chinese controls in-house exome database from Novogene Bioinformatics Institute (Beijing, China). After excluding synonymous variants, potential pathogenic impacts caused by non-synonymous variants located in exonic regions and in splicing sites were predicted using the following four tools: Polymorphism Phenotyping v2 (PolyPhen-2) (http://genetics.bwh.harvard.edu/pph2/), Sorting Intolerant from Tolerant (SIFT) (http://sift.jcvi.org/), MutationTaster software (http://www.mutationtaster.org/), and Combined Annotation Dependent Depletion (CADD) algorithm (http://cadd.gs.washington.edu/score). According to the American College of Medical Genetics and Genomics (ACMG) guidelines for variants interpretation, variants for STGD1 or STGD1-like phenotypes were classified as pathogenic, potentially pathogenic, unknown clinical significance, likely benign, or benign [25].

### Sanger sequencing

Potential variants identified by whole exome sequencing in the genes associated with STGD1 or STGD1-like phenotypes were selected for subsequent Sanger sequencing in the proband, available family members, and 100 unaffected individuals. Sequence results were then analyzed with Chromas software (version 2.01, Technelysium Pty Ltd., South Brisbane, Australia). Co-segregation of potential pathogenic variants with this disease phenotype was also analyzed. Primers were designed around potential missense variants with Primer3 software (http://primer3.ut.ee/) based on the genome sequences of human reference genome UCSC (GRCh37/hg19). These primers were then synthesized in Sangon Biotech (Shanghai) Co., Ltd. (Shanghai, China). Primer sequences for PCR and Sanger sequencing were as follows: 5′-ACCCAAGTATGGCCCGTCCA-3′, 5′-TCCCATCCATCTGTTGCAGG-3′; and 5′-CAACCCACACTGGGTGTTCT-3′, 5′-TGCATCCCCTAGATTTGGAG-3′.

### Conservative analysis and structure modeling

Multiple sequence alignments were performed using the online Clustal Omega tool (http://www.ebi.ac.uk/Tools/msa/clustalo/) in seven different species. Predictions for structures of wild-type and mutant proteins were performed by online SWISS-MODEL tool (http://www.swissmodel.expasy.org). Structures were visualized using PyMOL software (version 1.5, Schrödinger, LLC, Portland, U.S.A.) [7].

## Results

### Clinical findings

**Figure 1:**
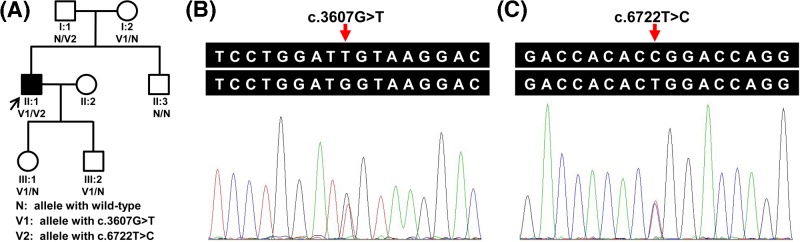
Identified variants of *ABCA4* in the STGD1 family (**A**) A pedigree of a Han Chinese family with STGD1. Squares and circles represent males and females, respectively. The darkened symbol indicates the affected member, and the open symbols indicate the unaffected members. The patient above the arrow is the proband of this family. (**B**,**C**) Sanger sequencing verified that the proband (II:1) harbored the compound heterozygous variants (c.3607G>T and c.6722T>C).

**Figure 2:**
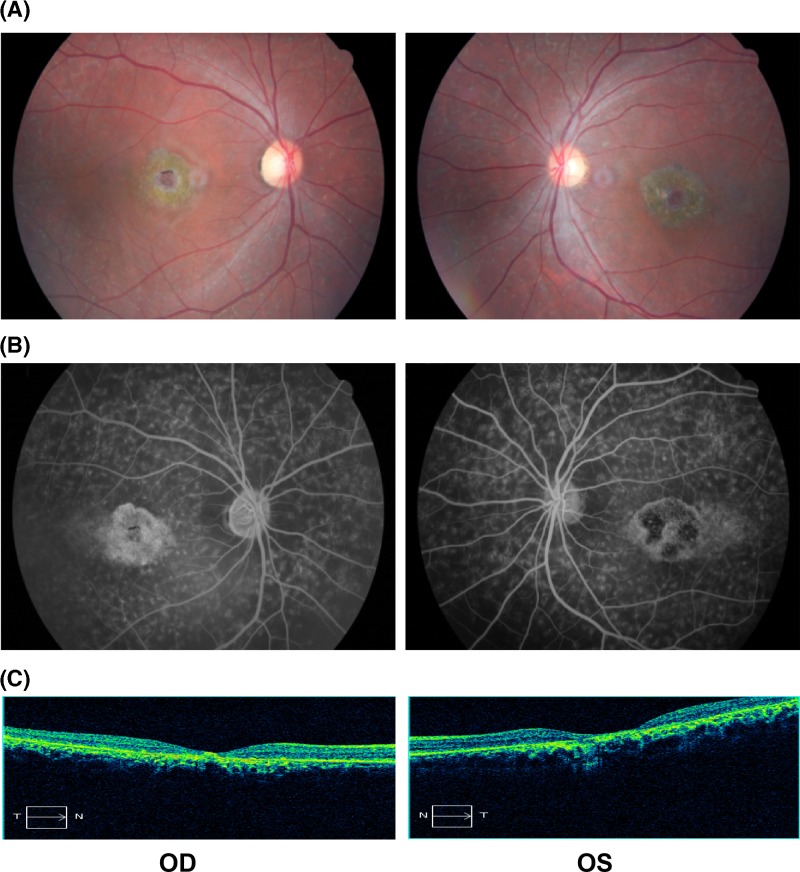
Clinical characteristics of the proband (**A**) Fundus photographs showed some pigment mottling, a beaten-bronze appearance in the macular area, and yellowish flecks in bilateral maculae. (**B**) FFA revealed extension of hyper-fluorescent flecks into the mid-peripheral retina, and formation of fluorescence blocking in the maculae due to pigment mottling. (**C**) OCT showed hyper-reflective deposition between the RPE layer and the photoreceptors outer segments, retinal outer layers attenuation, and choroidal reflectivity increment. Abbreviations: OD, right eye; OS, left eye.

Seven members of a three-generation family with STGD1 were presented in the present study (Figure 1A). The proband (II:1), a 35-year-old man of Han Chinese, presented poor visual acuity at 6 years of age. His BCVA was 20/66 (right) and 20/200 (left). Ophthalmoscopic examination of the proband showed bilateral macular atrophy, and photoreceptor and RPE cells degeneration. Fundus imaging demonstrated some pigment mottling, a beaten-bronze appearance in the macular area, and yellowish flecks in bilateral maculae (Figure 2A). FFA revealed extension of hyper-fluorescent flecks into the mid-peripheral retina, and formation of fluorescence blocking in the macular area due to pigment mottling (Figure 2B). OCT showed hyper-reflective deposition between the RPE layer and the photoreceptor outer segments, retinal outer layer attenuation, and choroidal reflectivity increment (Figure 2C). As a result, he was diagnosed with STGD1. His 60-year-old mother was diagnosed with visual impairment due to myopia, but had no other ocular abnormalities. His father, brother, and offsprings manifested no obvious ocular problems.

### Whole exome sequencing and pathogenic variants validation

**Figure 3:**
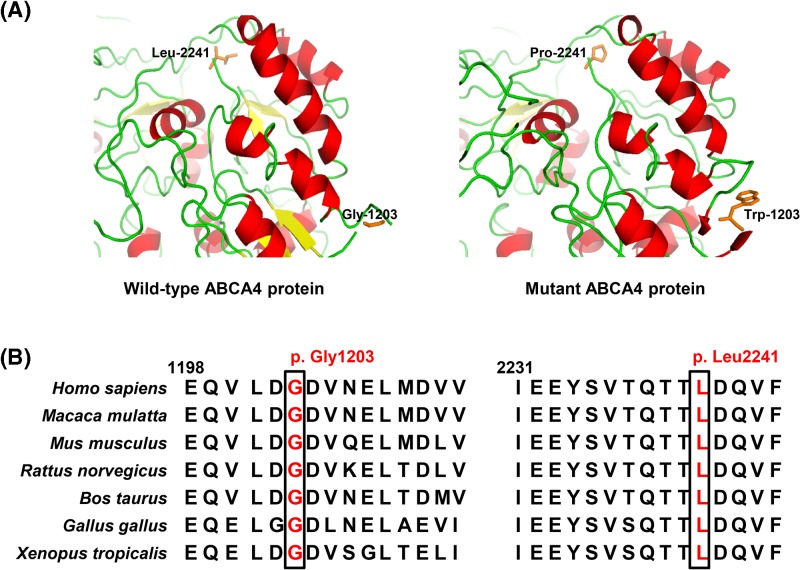
Structure prediction and conservation analysis of wild-type and mutant ABCA4 proteins (**A**) Predicted structures of wild-type and mutant ABCA4 proteins showed that the changes in glycine to tryptophan at position 1203 and leucine to proline at position 2241 caused conformational alteration. The crystal structure of the ABCA1 protein (PDB ID: 5XJY, 51.97% sequence identity) was used as template. (**B**) Multiple sequence alignment in ABCA4 proteins from human to tropical clawed frog revealed that the glycine and leucine at mutant sites were both located within highly conserved regions, suggesting that these amino acids are crucial for normal protein function.

Target region average sequencing depth was 66.68×. A total of 98.8% regions covered by target sequences were 10× or more. Up to 22691 SNPs and 617 Indels were identified in coding regions and 2484 SNPs and 393 Indels in splice sites, which may have an effect on protein function. These variants were further filtered by 1000G_all, dbSNP147, ExAC_EAS, ESP6500_all, and in-house exome database from Novogene to assess possible pathogenic variants in the proband. The rest of potential deleterious variants were screened out by further analysis with PolyPhen-2, SIFT, MutationTaster, and CADD. In all known disease-causing genes associated with STGD1 or STGD1-like phenotypes, only two compound heterozygous *ABCA4* variants, c.3607G>T (p.(Gly1203Trp)) in exon 24 and c.6722T>C (p.(Leu2241Pro)) in exon 48, were found in the proband. According to the variant classification guidelines of ACMG, these two variants are categorized as likely pathogenic variants. Sanger sequencing further verified both variants in the proband (II:1, Figure 1B,C). In the pedigree, the proband’s 66-year-old unaffected father (I:1) possessed the heterozygous c.6722T>C variant, and the 60-year-old myopic mother (I:2) had the heterozygous c.3607G>T variant. The 8-year-old unaffected daughter (III:1) and 6-year-old son (III:2) harbored only the c.3607G>T variant. The proband’s 34-year-old brother (II:3) did not carry these variants. These indicated that the compound heterozygous variants (c.3607G>T and c.6722T>C) co-segregated with this disease in this family. Both variants were absent from the 100 Han Chinese unaffected controls. Structural modeling revealed the conformational alteration after the glycine at residue 1203 (Gly-1203) and leucine at residue 2241 (Leu-2241) changed into tryptophan (Trp-1203) and proline (Pro-2241), separately (Figure 3A). Both residues at mutant sites (p.Gly1203 and p.Leu2241) are highly conserved in the ABCA4 proteins from human to tropical clawed frog (Figure 3B), suggesting that the two variants are likely pathogenic.

## Discussion

STGD1 is a common AR hereditary juvenile macular degeneration resulting from immoderate lipofuscin accumulation in RPE [21,26]. Patients often have visual acuity loss during the first or second decade of life as a result of macular atrophy and yellowish lipofuscin flecks deposition in the RPE [1]. In the present study, STGD1 was clinically diagnosed with the symptoms. The proband onset age was at 6 years and accompanied with central visual acuity loss. FFA and OCT revealed an excessive accumulation of lipofuscin flecks within the RPE and a dramatic decrease in retinal thickness in the central foveola. These indicated a significant influence on photoreceptor structure integrity. No other member of this family has obvious ocular abnormalities except for the myopia in his mother (Table 1).

**Table 1:** Clinical and genetic characteristics of available members in this family

Family member	Age (years)	Sex	BCVA (OD, OS)	Fundus	OCT	Clinical diagnosis	Genotype
I:1	66	Male	20/20, 20/33	Normal	Normal	Asymptomatic	N/V2
I:2	60	Female	20/250, 20/133	Normal	Normal	Short-sightedness	V1/N
II:1	35	Male	20/66, 20/200	Macular atrophy, yellowish flecks in RPE	Hyper-reflective deposition, retinal outer layers attenuation	STGD1	V1/V2
II:3	34	Male	20/20, 20/33	Normal	Normal	Asymptomatic	N/N
III:1	8	Female	20/20, 20/20	Normal	N/A	Asymptomatic	V1/N
III:2	6	Male	20/20, 20/25	Normal	N/A	Asymptomatic	V1/N

Abbreviations: N, allele with wild-type; N/A, not available; OD, right eye; OS, left eye; V1, allele with c.3607G>T; V2, allele with c.6722T>C.

Genotypic multiplicity and phenotypic variability widely exist in inherited retinal disorders. Mutations in the *ELOVL4, PROM1, PRPH2, BEST1*, and *CRB1* have been reported to be responsible for STGD1-like disease in previous studies [9–12]. In addition to STGD1, mutations in *ABCA4* result in a spectrum of related retinal degenerative diseases with variable clinical phenotypes, including retinitis pigmentosa-19, age-related macular degeneration, and cone-rod dystrophy [27,28]. Due to high polymorphic heterogeneity and large size of *ABCA4*, as well as misdiagnosis of STGD1, time and cost remain prohibitive for large-scale documented STGD1-related mutation analysis using common Sanger sequencing [29,30]. Whole exome sequencing provides an effective approach to screen pathogenic mutations in exonic regions of protein-coding genes at a single nucleotide resolution in Mendelian disorders such as inherited retinal disorders [18,31]. In the present study, whole exome sequencing combined with Sanger sequencing was performed to screen genetic pathogenic causes in this Han Chinese family with STGD1. Two compound heterozygous variants, c.3607G>T (p.(Gly1203Trp)) and c.6722T>C (p.(Leu2241Pro)), in *ABCA4* associated with STGD1 phenotype were identified in this family. The c.3607G>T variant located at a splice site where a c.3607G>A variant had been reported to affect splicing *in vitro* [32] was also predicted to most likely have an effect on the splicing of the RNA by the Alamut Visual software (Interactive Biosoftware, Rouen, France) and Berkeley Drosophila Genome Project (http://www.fruitfly.org/seq_tools/splice.html). These two variants are categorized as likely pathogenic variants in accordance with the variant classification guidelines of ACMG. The effects on protein function of the two missense variants (p.(Gly1203Trp) and p.(Leu2241Pro)) were predicted to be deleterious by online prediction programs (PolyPhen-2, SIFT, MutationTaster, and CADD) (Table 2). Four family members (I:1, I:2, III:1, and III:2) with heterozygous *ABCA4* variant (p.(Gly1203Trp) or p.(Leu2241Pro)) were asymptomatic, which may be due to reserving a partly normal ABCA4 protein, supporting that the compound heterozygous variants (p.(Gly1203Trp) and p.(Leu2241Pro)) were responsible for the STGD1 phenotype in this family.

**Table 2:** Identification of *ABCA4* variants in the proband

Categories	Variant 1	Variant 2
Chromosomal location (change)	Chr1:94505599 (C→A)	Chr1:94463424 (A→G)
Exon	24	48
Nucleotide change	c.3607G>T	c.6722T>C
Amino acid change	p.(Gly1203Trp)	p.(Leu2241Pro)
Mode	Heterozygous	Heterozygous
Variant type	Missense	Missense
dbSNP147	Novel	Novel
1000G_all	Novel	Novel
ExAC_EAS	Novel	Novel
ESP6500_all	Novel	Novel
Novogene in-house exome database	Novel	Novel
PolyPhen-2_HumVar (score, prediction)	0.883, possibly damaging	0.972, probably damaging
PolyPhen-2_HumDiv (score, prediction)	0.989, probably damaging	0.999, probably damaging
SIFT (score, prediction)	0.028, damaging	0.006, damaging
MutationTaster (probability value, prediction)	1, disease causing	1, disease causing
CADD v1.3 (phred-score, prediction)*	35, deleterious	28.7, deleterious
Maternal allele	Yes	/
Paternal allele	/	Yes

Abbreviations: ESP6500_all, Exome Sequencing Project 6500_all; ExAC_EAS, Exome Aggregation Consortium_East Asian; 1000G_all, 1000 Genomes Project_all.

* Variant is regarded as deleterious when its phred-score is greater than 15 [41].

The *ABCA4*, located at chromosome 1p22.1, includes 50 exons and expresses a 2273-amino acid glycoprotein with a molecular mass of 256 kDa, located in outer segments of cone and rod photoreceptors [8,14]. *ABCA4* mutations usually result in decrease or loss of retinal-activated ATPase activity and/or NRPE flipping activity, as well as protein mislocalization caused by misfolding in photoreceptor cells [3]. In the present study, amino acid alteration of the hydrophilic glycine residue to the hydrophobic tryptophan residue at position 1203 (p.(Gly1203Trp)) may impact the function or tertiary structure. This alteration located in the cytoplasmic domain 1 (although often called nucleotide binding domain 1) probably covers ABCA4 protein binding sites due to hydrophobic residues mainly tending to locate on molecule surfaces [13,33]. Interestingly, a p.(Gly1203Arg) missense mutation has been reported in an Italian patient with STGD1 [34]. These suggest the functional importance of the hydrophilic glycine residue. However, the change of leucine residue to proline residue at position 2241 (p.(Leu2241Pro)) may impact retinal-activated ATPase activity on account of locating in the cytoplasmic domain 2 (although often called nucleotide binding domain 2) [13,33]. The p.(Leu2241Val) variant (c.6721C>G), a mutation at the same amino acid position, has been reported in a German individual with STGD1 [35]. This indicates the importance of this amino acid in the intracellular nucleotide binding domain. Glycine residue at position 1203 and leucine residue at position 2241 are both strictly conserved in ABCA4 proteins from human to tropical clawed frog by conservation analysis (Figure 3B), suggesting that these amino acids are crucial for normal protein function. Intriguingly, the p.(Gly863Ala) and the p.(Asn965Ser) mutations associated with STGD1 in the cytoplasmic domain 1 where the p.(Gly1203Trp) mutation is located were reported to display ∼18 and 37% of wild-type NRPE flipping activity and result in ATPase activity decrease [36]. Similarly, the p.(Leu2027Phe) and the p.(Arg2077Trp) mutations in the cytoplasmic domain 2 where the p.(Leu2241Pro) mutation is located were found to change the subcellular localization, and were devoid of NRPE flipping activity and ATPase activity [37]. These findings further hint that p.(Gly1203Trp) and p.(Leu2241Pro) variants possibly cause the similar reduction in NRPE flipping activity and ATPase activity, which promotes the progression of this disorder. However, the shortcoming of the present study is lack of evidence to support the variants’ pathogenicity in functional experiments. Identification of novel disease-causing mutations in more families and performing functional studies can be of great help to reveal the pathogenic mechanism underlying the ATP-dependent substrate transport of this disorder.

Approximately, 90–95% of STGD1 patients harbor loss-of-function *ABCA4* mutations and are inherited as an AR form [1]. There are currently no effective remedies for STGD1-caused vision loss. Gene therapy is a promising therapeutic approach for treatment or prevention of a genetic defect disease by providing a therapeutic effect gene. *Abca4* knockout mice revealed some clinical manifestations related to STGD1 including delayed dark adaptation, RPE layer thickening, outer retinal layers thinning, lipofuscin accumulation in RPE cells, and photoreceptor degeneration [38]. Amazingly, in *Abca4* knockout mice, delivering mouse *Abca4* with recombinant adeno-associated viral vectors and human *ABCA4* with recombinant lentivirus vectors into RPE and photoreceptors cells could trigger dark adaptation recovery, RPE layer thinning, and retinal lipofuscin deposition decrease [14,38,39]. New genome editing treatment approaches such as CRISPR/Cas9 system could specifically target a genetic defect on a chromosome and replace it with a normal DNA sequence rather than an entire gene [40]. This shows its potential application as a treatment for STGD1 and holds great promise for treating *ABCA4*-associated hereditary retinal diseases like STGD1.

Taken together, the present study identified two novel potentially pathogenic variants in a compound heterozygous state, c.3607G>T (p.(Gly1203Trp)) and c.6722T>C (p.(Leu2241Pro)), in *ABCA4* responsible for a STGD1 phenotype in a three-generation Han Chinese family and broadened the mutational spectrum of *ABCA4* in STGD1. A combination of whole exome sequencing and Sanger sequencing is likely to be a time-saving and cost-efficient approach to screen pathogenic variants in genetic disorders caused by sizable genes, as well as avoiding misdiagnosis. These results perhaps refine genetic counseling and *ABCA4*-targetted treatments for families affected by STGD1.

## Abbreviations

*ABCA4*: ATP binding cassette subfamily A member 4 gene
ACMG: American College of Medical Genetics and Genomics
AR: autosomal recessive
BCVA: best corrected visual acuity
*BEST1*: bestrophin 1 gene
BWA: Burrows–Wheeler Aligner
CADD: Combined Annotation Dependent Depletion
*CRB1*: crumbs 1 cell polarity complex component gene
dbSNP147: Single Nucleotide Polymorphism Database 147
*ELOVL4*: ELOVL fatty acid elongase 4 gene
ESP6500_all: Exome Sequencing Project 6500_all
ExAC_EAS: Exome Aggregation Consortium_East Asian
FFA: fundus fluorescein angiography
gDNA: genomic DNA
Indel: insertion/deletion
NRPE: N-retinylidene-phosphatidylethanolamine
OCT: optical coherence tomography
PolyPhen-2: Polymorphism Phenotyping v2
*PROM1*: prominin 1 gene
*PRPH2*: peripherin 2 gene
RPE: retinal pigment epithelium
SIFT: Sorting Intolerant from Tolerant
SNP: single nucleotide polymorphism
STGD1: Stargardt disease
1000G_all: 1000 Genomes Project_all
